# Significant improvements in pain after a six-week physiotherapist-led exercise and education intervention, in patients with osteoarthritis awaiting arthroplasty, in South Africa: a randomised controlled trial

**DOI:** 10.1186/s12891-016-1088-6

**Published:** 2016-05-27

**Authors:** M. M. Saw, T. Kruger-Jakins, N. Edries, R. Parker

**Affiliations:** Physiotherapy department, Tygerberg Hospital, Cape Town, South Africa; ICAS South Africa, Musculoskeletal Health, Johannesburg, South Africa; Department of Health & Rehab Sciences, Faculty of Health Sciences, University of Cape Town, Cape Town, South Africa

**Keywords:** Hip/knee osteoarthritis, Arthroplasty/joint replacement, Waiting list, Physiotherapy, Exercise, Education, Chronic pain

## Abstract

**Background:**

A major challenge facing those with late stage osteoarthritis is delayed surgery due to waiting lists. In South Africa patients wait years for a hip/knee arthroplasty. Affected patients require effective management to address their pain, especially while awaiting surgery. Existing literature is mostly available from high income countries exploring effects of interventions during short waiting periods. Research is warranted in low income countries where long waiting periods are common. This study explored the effects of a six-week physiotherapist-led exercise and education intervention on pain in this population.

**Methods:**

A randomised controlled trial was performed at two public hospitals in South Africa. Ethical approval and informed consent was obtained. 74 participants from arthroplasty waiting lists were randomly allocated to an intervention (*n* = 35) or control group (*n* = 39). The intervention included six physiotherapist-led group-based sessions (two hours/week of education, exercise and relaxation). The control group received usual care. Data collection was conducted by blinded physiotherapists at baseline, week six, 12 and month six. The primary outcome was pain, measured by the Brief Pain Inventory. Additionally, participants completed an open-ended questionnaire at month six, to gain insight regarding the intervention. Analysis was by intention to treat using two-way analysis of variance and post-hoc Tukey comparisons. Answers to subjective questions were analysed according to common themes that emerged.

**Results:**

The intervention group had significant improvements compared with the control group with moderate to large effect sizes (ES) on pain severity [week 6: *p* < 0.01, ES = 0.94, 95 % CI (0.45,1.41), month 6: *p* = 0.02. ES = 0.74, 95 % CI (0.26,1.2)] and moderate to large effects on pain interference [week 6: *p* < 0.01, ES = 1.2, 95 % CI (0.70,1.69), week 12: *p* = 0.04, ES = 0.68, 95 % CI (0.20,1.14), month 6: *p* < 0.01, ES = 0.98, 95 % CI (0.49,1.45)]. 53 % of participants reported that the intervention improved their pain.

**Conclusions:**

The intervention resulted in sustained significant improvements in pain severity and interference in patients with hip/knee osteoarthritis, awaiting arthroplasty compared with a control group. Additionally, participants’ individual feedback supported observed significant improvements in pain. Such an intervention appears to be effective in managing pain in this population and should be incorporated into practice for appropriate patients. Further research is being conducted to explore long term and postoperative outcomes.

**Clinical trial registration:**

Pan African Clinical Trial Registry, PACTR201409000885765, PACTR201507001186115.

**Electronic supplementary material:**

The online version of this article (doi:10.1186/s12891-016-1088-6) contains supplementary material, which is available to authorized users.

## Background

Osteoarthritis (OA) is known as the most common joint disease worldwide [1]. The prevalence of OA continues to rise exponentially in both high and low income countries, including South Africa (SA) [2]. The statistics from the Global Burden of Disease studies of 2010 reported the age-standardised prevalence rates of knee OA in Southern Sub Saharan Africa to be 3.1 % [95 % confidence interval (CI) 2.1, 4.5] in males and 5.2 % (95 % CI 3.5, 7.4) in females, with lower prevalence for those with hip OA (males: 0.6 %, 95 % CI 0.5, 0.7. Females: 0.8 %, (95 % CI 0.6, 0.9) [2]. This increasing prevalence is partlydue to an increasingly aged population, decreased physical activity and obesity [2–4]. The burden of OA leads to a large portion of the older population suffering with severe pain on a daily basis [5, 6]. Due to the chronicity and complexity of OA, effective management is necessary at all stages of the condition to manage this population’s pain.

As insight into this field of research is constantlyimproving, many evidence-based guidelines have beenpublished for the management of hip and knee OA [7–14]. The evidence-based management for OA begins with non-pharmacological methods of education, exercise and weight control. Pharmacological agents are subsequently added as required, in combination with the above first-line management. Finally, surgery is indicated for those with late stage OA who do not respond to conservative treatment methods [13, 14].

A major concern internationally is the limited accessibility of the recommended management options, especially in the public health system [4]. This is true for many individuals in SA where first line management options are not readily accessible and delays in surgery are experienced as a result of long waiting lists [15].

Waiting lists in secondary and tertiary health facilities in SA have been reported to range from one to eight years depending on the severity of the condition and the resources available [15]. Waiting for surgery for six month or longer is reported to have various negativeimpacts on the individual including worse pain, function and quality of life, adding to the burden of OA [16, 17].

### Evidence for an exercise and education intervention

It is proposed that combining first line management recommendations for OA of exercise and education could provide a patient centred approach to ongoing management while awaiting arthroplasty [18, 19]. While exercise interventions alone have shown no effects on pain or function in persons awaiting total knee replacement [20] and only low to moderate effects on those awaiting total hip replacement [20, 21], education and exercise delivered by programmes such as the ESCAPE programme [22] and/or arthritis self-management programme [23] have been reported to be an effective method of not only improving knowledge, but also prolonging the belief that exercise is beneficial and achieving desired changes in exercise habits [22]. Asevidence suggests, instead of merely educating on the importance of exercise, interventions should include both educational and active participatory exercise components [23, 24]. To further expand on this it is anticipated that by combining both aspects, the optimal benefits of these two management options could be achieved by allowing the person to incorporate new knowledge from the educational component into practice and implement the advised changes in beliefs and behaviours, such as exercise, in order to experience the benefits first hand [22–27]. Previous studies have shown benefits from various exercise and education interventions in persons with hip and knee OA; ranging from improvements in pain [24, 28–30], function [24, 28–31], self-efficacy [28, 29, 32, 33], and exercise participation [28, 33]. Despite literature supporting the use of combined interventions in persons with OA, studies specific to those with severe OA and awaiting surgery for extended periods such as the population in SA, are scarce.

To the authors’ knowledge, no such studies have been performed in the SA population and the above mentioned studies performed in higher income countries with more accessible resources and shorter waiting times cannot be generalised to the SA population.

Therefore, in response to the increasing burden of OA and long waiting lists for hip and knee arthroplasty in the public health system in SA, research was warranted to establish the effects of a pre-operative exercise and education intervention on pain, for patients with OA awaiting arthroplasty. Additionally, we aimed to establish whether any changes in pain were supported by individual responses when interviewed about participating in the intervention…

## Methods

### Study design

A randomised controlled trial was performed and is reported according to CONSORT (Consolidation of Standards of Reporting Trials) guidelines.

### Ethical Approval

The University of Cape Town Human Research and Ethics Committee granted approval for this study (Ref 378-2013, 492-2013). The clinical trial was registered with the Pan African Clinical Trial Registry (PACTR201409000885765, PACTR201507001186115).

### Participants

The population of interest was patients diagnosed with OA in SA. The sampling frame included patients who had been placed on a waiting list to receive a hip/knee arthroplasty, specifically in the public health system inthe Western Cape and Gauteng provinces. Principle investigators were given access to waiting lists at Tygerberg and Helen Joseph Hospital. Participants were required to be on the waiting list for a minimumof three months to allow for necessary stabilisation to any new medications that may have been prescribed or adjustments to their treatment when the patient was first put onto the list. Inclusion criteria were as follows: a willingness to commit to the study, aged between 50 – 70 years, diagnosed with OA of the hip/knee, literate in English,Afrikaans, isiXhosa or isiZulu. Exclusion criteria were any cognitive impairment, as reported in the medical records, previous trauma/surgery to the unaffected leg, deemednot eligible for exercise as per the American College of Sports Medicine (ACSM) guidelines for exercise prescription. Reasons for exclusion according to theACSM included previous cardiac conditions or surgery,uncontrolled diabetes or asthma [34]. Additionally those who had previously taken part in a six-week programme aimed at improving self-efficacy and management were excluded.

Since pain was the primary outcome measure in this study; a change in pain severity was selected to determine the required sample size using Brief Pain Inventory (BPI) data from a previous study on pain in people with OA [35]. Calculations used a smallest meaningful difference in pain scores of 3 (on a scale of 0 to 10 on the BPI), and a standard deviation of 2.49 [35]. Statistical significance was accepted as *p* < 0.05 with a medium to large effect size (ES > 0.5) accepted as meaningful. It was calculated that samples of 26, 36, and 45 participants in total would provide 80 %, 90 % and 95 % statistical power for change in pain respectively.

To allow for attrition at recruitment or during the study, a higher target sample size was preferred and recruitment was aimed at a maximum of 48 participants at each site to make up 2 groups of 24 (a maximum of 96 participants across both sites).

### Recruitment procedure

**Fig. 1:**
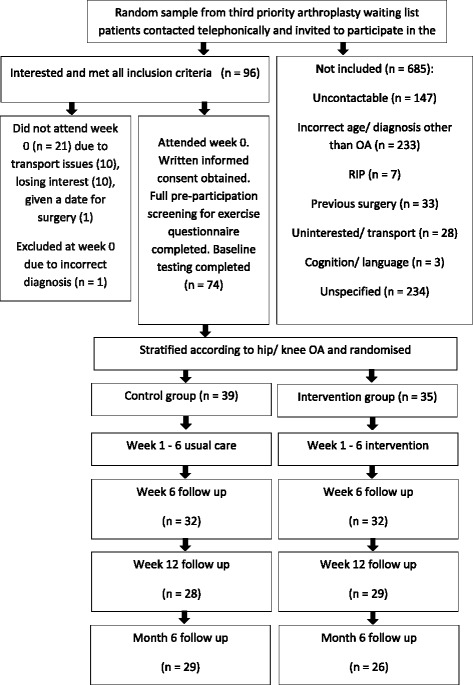
Recruitment and randomisation process

The waiting lists at both facilities combined wereapproximately 4300 patients requiring arthroplasty for various reasons, including OA. Due to limited research personnel in the public health setting in SA and notbeing able to contact all patients, a random sample of 20 % of the total waiting list (*n* = 781) was drawn forlogistical reasons, using random number selection in Microsoft Excel. This was done to ensure patients had a fairer chance of being contacted, rather than contacting patients from the top of the list down. Diagnosis was made by the orthopaedic surgeon at the first clinic visit when the patient was placed on the waiting list from clinical history, physical examination and radiographic images according to American College of Rheumatology (ACR) clinical and radiological criteria. Those in the sample that met inclusion criteria were contacted telephonically by the primary investigators, to inform them of the nature of the study and to invite them to participate, if eligible. A large number of participants (*n* = 685) were unreachable and/or excluded from participating for various reason such as incorrect age/diagnosis other than OA according to the waiting list information, previous surgery, not interested, transport issues (Fig. 1). Thus 96 participants were invited to attend baseline testing, 21 did not arrive due to losing interest or transport issues and 1 was excluded due to incorrect diagnosis, therefore 74 arrived and were eligible to partake.

The first 10 participants’ baseline meeting was utilised to pilot the baseline procedures of providing all relevant information to the participants, obtaining informedconsent, completing demographic information and completing baseline outcome measures.

This took place at the setting intended for the study where outcome measures and the intervention took place, with the presence of the principle investigator for the information and informed consent signing and the blinded physiotherapist research assistant (RA) for completing the outcome measures, at each hospital. Piloting went smoothly as participants were able to complete outcome measures with the assistance of the RA without issues, it was noted that the time allocated to eachsession of data collection was sufficient and no changes were needed to the methods of collecting data for theprimary outcome measure. Thereafter, the remaining 64 participants gave written informed consent anddemographic information (including age, gender, educational level, employment status, joints affected, BMI, co-morbidities and waiting time for surgery) was recorded with the help of the research assistant. The participants completed the outcome measures, assisted by the research assistant with a translator where necessary.

**Fig. 2:**
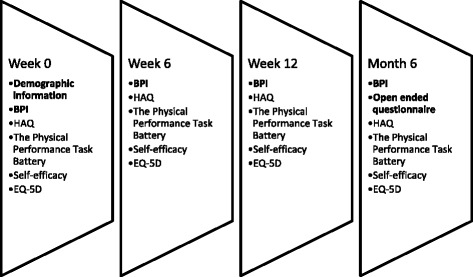
Data collection process

The participants were stratified, according to OA of the hip or knee and then randomly assigned into either the experimental or control group, by means of random number allocation. This was performed by the twoprimary investigators. Those selected for the control group (*n* = 39) were contacted telephonically in the week that followed baseline assessment and were instructed to continue receiving their usual care, determined by their primary doctor, while awaiting surgery. The experimental group (*n* = 35) were contacted telephonically and were requested to begin the six-week education andexercise intervention commencing the following week (Fig. 1). Follow up measures were taken at week sixfollowing the intervention, and again at week 12 and month six, which concluded the study (Fig. 2).

### Intervention

It was proposed that this intervention has the potential to manage, yet probably not cure pain in this chronic condition, as it is based on the International Classification of Functioning, Disability and Health’s (ICF) understanding of health [26, 33]. This framework targets physiologicalimpairments (pain, muscle weakness, joint stiffness, and low fitness levels), personal factors (knowledge, health beliefs, self-efficacy, and stress management) and behaviours during daily activities (avoidance of movement or exercise, eating and sleeping habits, goal setting) [26, 33]. The intervention comprised of a six week physiotherapist-led education and exercise programme which was developed from previously used programmes [25, 33, 35–38].

This multi-faceted approach addresses the participant holistically by means of education (including pain neuroscience education), self-management strategies and an active exercise component. The intervention approach and content allows for accommodation of participants with OA of both hip and knee. The intervention took place in an out-patient setting at both hospitals and wasled by the principle investigators, two qualified physiotherapists who had been trained in the respected field and who could deliver the programme in both English andAfrikaans. IsiXhosa and isiZulu translators were present for intervention sessions. The same physiotherapists led the six sessions to ensure content and delivery of the intervention remained constant. It took place for a maximum of two hours, on a weekly basis for six successive weeks, in groups of no more than 12 participants. Each two hour session included an educational component of approximately an hour, an exercise component of 20 to 30 min and a relaxation session of 10 min.

### Educational component

The educational topics covered are outlined in Table 1. The model of delivery of this intervention is based on the principles of social learning and cognitive behaviouralapproaches [25, 27, 32, 39, 40]. The educational component was aimed at increasing knowledge and understanding of OA, pain neuroscience, activity and related topics affected by their condition. Important topics such as self-management skills, problem solving, goal setting, coping mechanisms, stress management, and pacing were discussed to enable the participant in self-management [27]. Each participant received a “Living with Osteoarthritis” workbook (in their preferred language) on the content of each educational session, which they were encouraged to work through each week [41].

**Table 1:** Educational content covered on a weekly basis during the intervention

Topic for the week	Content covered
Week 1: Osteoarthritis, self-management and exercise	Pathology of OA. What is meant by “self-management”? Self-management steps.Action plans, goal setting. Exercise dos and don’ts. Types of exercise, steps tosuccess with exercise. An exercise routine.
Week 2: Managing common symptoms	Physiology of acute and chronic pain. Pain and flare ups of pain. Swelling.Joint protection, assistive devices. Pacing and activity/resting cycles.Fatigue, frustration, isolation.
Week 3: Stress management	What is stress? Managing stress. Sleep management. Communication withyour health carer. Relaxation skills.
Week 4: Eating well	Balanced nutrition. Dealing with barriers to eating well. Food safety,weight loss benefits.
Week 5: Medication and disease related problem solving	Making informed treatment decisions. Appropriate use of medications.Link between a healthy lifestyle, good nutrition and exercise. Communicatingeffectively with family, friends, and health professionals with regardsto your problems.
Week 6: Continuing as a successful self-manager	Recap of key components of successful self-managing, Action planning forthe future. Reflection on changes.

### Exercise component

The exercise component allowed participants to apply what was learnt into actual behaviour changes [27]. It comprised of various stretching, light aerobic exercise and different lower limb muscle group strengtheningexercises [31]. Participants were required to set exercise and activity goals on a weekly basis and to record thesein their workbooks. The exercise component commenced at low repetitions and intensity and was progressed weekly from 20 min in duration, increasing time by 10 % andintensity as appropriate, depending on each participant’s individual ability. As the group size was small, safetyduring exercise and appropriate progression was possible despite being in a group [23]. The detailed exercise programme is described in detail (see Table 1). The intervention concluded with a relaxation session led by the physiotherapist facilitating various relaxation visualisations, an example of which is included in the workbook. Although relaxation techniques are not included in the recommended management of OA, it is widely used in chronic pain conditions and is seen as a helpful self-management skill [42, 43].

Attendance for the intervention was recorded and monitored weekly. If a participant was absent for asession of the intervention, the participant was contacted telephonically and asked the reason for absenteeism and encouraged to attend the next session. The intervention took place as described above for six weeks.

### Instrumentation

The primary outcome measure was pain as measured by BPI [44]. The BPI is a short self-administered assessment tool for measuring the severity of pain and the effects thereof (out of 10) and is widely used in both clinical and research environments [45]. To address shortcomings found with other pain scales that neglect pain’s characteristic as a variable factor, the BPI evaluates pain severity on the person’s “worst”, “least”, “average” and “now” pain over the last 24 h instead of one score forintensity of pain during different tasks. A pain severity score is calculated as an average of these four scores.Pain interference is scored by evaluating the effect of pain on an activity sub-dimension (walking, generalactivity and work) and four other aspects of life (relationships with others, enjoyment of life, mood, forming the affective sub dimension, and sleep) [45] (see Additional file 1 for the BPI). These two dimensions (severity and functioning) are the first two dimensions, described by the Initiative on Methods, Measurement, and Pain Assessment in Clinical Trials (IMMPACT) panel, which should be included in all research dealing with chronic pain evaluation [46, 47].

Psychometric testing has been performed on the BPI’s use in OA [35, 48] and its’ use in the assessment of pain to distinctly depict pain intensity and its effects on function is supported [49–51]. The BPI has high levels of testre-test reliability [35, 52–54] and internal consistency in various languages and does not display cultural bias [55, 56]. Of particular interest to this study, the BPI has been used in studies of OA which reported good internal consistencies; particularly for the subscale of pain interference [57, 58].

The BPI has previously been translated into Afrikaans [59] as well as isiXhosa [40]. The IMMPACT panel has specifically singled out the pain interference scale from the BPI as a recommended scale to use for assessment of pain-related functional impairment [60].

Besides the BPI, secondary measures of disability [Health Assessment Questionnaire (HAQ)] [61], function (The Physical Performance Task Battery) [62], self-efficacy (The Self-Efficacy for Managing Chronic Disease 6-Item Scale) [63] and health related quality of life (EQ-5D) [64] were taken at all follow up intervals (Fig. 2).

In addition, the experimental group were asked to complete an open ended questionnaire at the six month follow up meeting. A simple questionnaire was constructed by the principle investigators to include seven open ended questions about the six-week intervention and workbook used. This questionnaire did not go through any process of validation. The questions are included in the Additional file 1. This was to gain some insight into the participants’ personal thoughts or feelings around the intervention and the individual effects thereof.

### Statistical analysis

The primary outcome measure of pain severity according to the BPI at baseline showed that data werenormally distributed [Week 0 BPI severity: *N* = 74, K-S p value > 0.20 (K-S p > 0.05)]. Therefore parametric statistics were used in the numerical analysis and Pearson Chi-squared (*χ*^2^) calculations were used for categorical data. However, waiting time was not normally distributed therefore non-parametric analysis was conducted and the Mann–Whitney *U* test was used for thesecalculations. Data was analysed using Statistica software(StatSoft, Inc. 2004. STATISTICA, Data Analysis Software System, Version 10. www.statsoft.com).

Statistical significance for the two main effects of groupand time, and the interaction (group x time) for the BPI was assessed using a two-way analysis of variance (ANOVA) with repeated measures. Tukey’s post hoc comparisons were performed where necessary to determine significance between groups at different time periods. Analysis was by intention to treat. Missingdata was managed by carrying forward the last observed measurement. All numerical data was presented as the mean (M) ± standard deviation (SD). Statistical significance was accepted as *p* < 0.05. Effect sizes (ES) werecalculated and reported on with 95 % CI where appropriate when there were significant differences between groups. Effect size was calculated as Cohen’s d [65] using the following formula:

$$ ES=\frac{experimental\  mean\  change - control\  mean\  change}{pooled\  baseline\ SD}. $$

Finally, a descriptive thematic analysis using an inductive approach was used to identify common themes from the answers to the open-ended questions. These themes were created to foster better understanding of theresponses to the questions related to the intervention.

## Results

Results are presented throughout for the entire sample (*N* = 74); the experimental group (*n* = 35) and the control group (*n* = 39). For the purposes of this manuscript no sub group analysis, according to OA hip or knee, have been reported on. The mean age for the sample was 60.72 years, SD = 5.54. The sample consisted of 14 males and 60 females, most of which (*n* = 59) were unemployed(receiving a pension or disability grant). Varied educational levels were noted; many participants had low levels of education of no higher than grade 7 (*n* = 16), with the majority having an educational level between grade 8 – 11 (*n* = 43). There were no significant differences between groups at baseline (*p* > 0.05) for age, gender, employment status or education.

The sample had a very high mean body mass index (BMI) value of 34.46, SD = 8.23 which is classified as class I obesity [66]. There were 30 participants who had hip OA, 32 with knee OA and 12 with both hip and knee OA. It was observed that the sample had a large number of co-morbidities present, with an average of 1.28 per participant.The most common condition present was hypertension (*n* = 29) as well as hypertension with diabetesmellitus (*n* = 13). Median waiting time for surgery when the study commenced was 1 years and 11 months, with a range of the shortest time on the waiting list being 3 months and the longest being 11 years. There were no significant differences between groups at baseline for time on the waiting list (U = 560.5, *p* = 0.18) or for BMI, joints affected or co-morbidities present (*p* > 0.05). According to baseline data, 40 participants were using analgesics, 28 were using analgesics and anti-inflammatories and six were not taking medication for pain relief; with the mean percentage pain relief obtained from medication at 47.92 %, SD = 25.56 %. According to the BPI, there were no significant differences in percentage pain relief obtained from medication between groups throughout the study [current effect: F(3, 177) = 0.63926, *p* = 0.59].

### Pain severity score

**Fig. 3:**
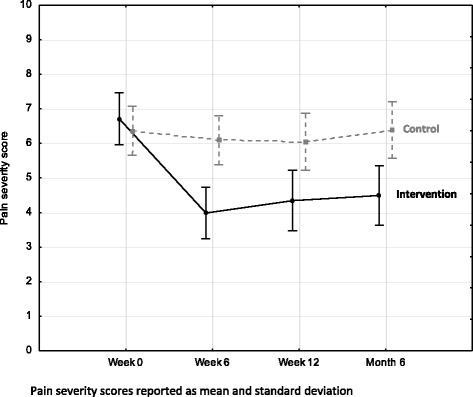
Pain severity score (*N* = 74)

At baseline, the pain severity scores demonstratedmoderate to severe pain in the whole sample (M = 6.53, SD = 2.29) (Table 2). Those who participated in the intervention group (IG) showed significant improvements in pain severity when compared to the control group (CG)with moderate to large effect sizes [current effect: F(3, 216) = 8.904, *p* < 0.01]. This was seen at week 6 by between-group differences of: M = 2.44, SD = 2.24, *p* < 0.01, ES = 0.94, 95 % CI (0.45, 1.41)] and at month 6: M = 2.24, SD = 2.39, *p* = 0.02, ES = 0.74, 95 % CI (0.26, 1.2) (Table 2 and Fig. 3).

**Table 2:** Brief Pain Inventory scores

	Experimental Group *n* = 35	Control Group *n* = 39	Between-group difference over time
	*Mean* ± SD (out of 10)	*Mean* ± SD (out of 10)	*Mean (95 % CI)*, ES (95 % CI)
Baseline Severity Interference	6.71 ± 2.32 6.75 ± 2.41	6.37 ± 2.16 6.44 ± 2.11	
Week 6 Severity Interference	3.99 ± 2.44* 3.13 ± 2.32**	6.09 ± 2.02 5.77 ± 2.07	2.44(0.6, 4.3), ES = 0.94(0.45, 1.41) 2.95(0.73, 5.2), ES = 1.2(0.7, 1.69)
Week 12 Severity Interference	4.34 ± 2.86 4.23 ± 2.72**	6.05 ± 2.34 5.95 ± 2.35	2.05(0.51, 3.6), ES = 0.66(0.18, 1.12) 2.03(0.5, 3.6), ES = 0.68(0.2, 1.14)
Month 6 Severity Interference	4.49 ± 2.85* 3.60 ± 2.63**	6.39 ± 2.30 5.98 ± 2.24	2.24(0.55, 3.9), ES = 0.74(0.26, 1.2) 2.69(0.66, 4.7), ES = 0.98(0.49, 1.45)

*indicates a significant improvement in pain severity of the experimental group compared to control group

**indicates a significant improvement in pain interference of the experimental compared to control group

### Pain interference score

**Fig. 4:**
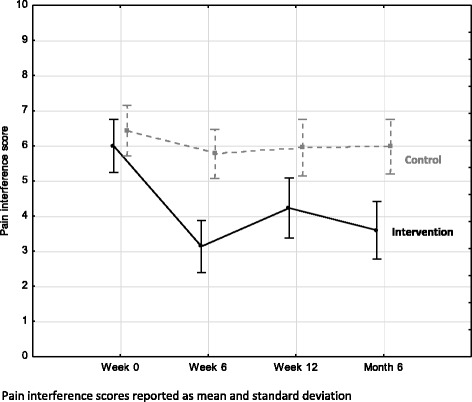
Pain interference score (*N* = 74)

Similarly, at baseline the sample had moderate to severe scores for pain interference (M = 6.23, SD = 2.25) (Table 2). Likewise, those who participated in the intervention group showed significant improvements in pain interference with moderate to large effect sizes when compared to the control group [Current effect: F(3, 216) = 6.85, *p* < 0.01]. This was observed at all intervals by mean between-group differences: week 6 M = 2.95, SD = 2.23, *p* < 0.01, ES = 1.2, 95 % CI (0.7, 1.69)], week 12 M = 2.03, SD = 2.41, *p* = 0.04,ES = 0.68, 95 % CI (0.2, 1.14)] and month 6 M = 2.69, SD = 2.18, *p* < 0.01, ES = 0.98, 95 % CI (0.49, 1.45) (Table 2 and Fig. 4).

### Open-ended questionnaire

Responses from 30 participants of the experimental group were collected as five did not complete this questionnaire. Themes were identified by analysing answers to questions one, two and five. The four themes that emerged were: pain relief, increased knowledge, improvement in function/activity, and personal/social benefits. Since this article’s main focus is on the effects of the intervention on pain, the answers relating to pain were analysed. Table 3 presents the answers given by participants showing the benefits experienced related to their pain.

**Table 3:** Subjective responses related to pain (*N* = 30)

Examples of the experimental group’s answers to open ended questionsone – five
Question 1: Did you find the 6 week course helpful to you in any way? If so how did it help you?
Participant V: “All the pain was gone” Participant A: “Much better with pain and sometimes without pain.” Participant N: “Yes, pain relief.” Participant R:”…and now I don’t have to rely on pain pills anymore.” Participant AA: “it gave me motivation again because my life was all about pain before. Now I use my workbook and it helps a lot” Participant CC: “…and it decreased my pain.” Participant XX: “…and to decrease pain.” Participant 36: “Yes the course was very helpful, but I am in painagain – then it was better, but not so much now” Participant 31: “Yes. I learned so much about my pain and how I can make changes for myself and make the pain better” Participant 11: “It was very helpful…I slept better and had nocramps – everything was much better.” Participant 9: “Absolutely 100 %. I got a lot of relief from my pain…” Participant 7: “My God – everything has changed. I don’t need operation anymore, I can work without pain…it takes my pain away – most ofthe time.” Participant 47: “…It helped me to manage my pain…”
Question 2: *What did you like about the course?*
Participant S: “I learnt how to put ice on my knee to relieve the pain.” Participant WW: “how to be more positive and to control pain.” Participant 5: “…learning about pain and what I can do to make it better.”
Question 5: What did you like about the workbook?
Participant R: “…also the section about pain management and not having to rely on pills” Participant 31: “Teaches us how to take care of ourselves, not to dependon medication…” Participant 35: “That it could teach me things about pain and healthyliving - I still read in it.”

Adverse responses were also noted in responses. One participant stated that although the intervention had been very helpful, the effect on her pain did not last. Another participant responded that accessibility had been a problem as elevators were not always operating well and wheelchairs were not readily available. The answers to the other four questions have not been included in this article as the majority of participants agreed in their answers in that there was nothing they did not like or nothing else that needed to be added to the intervention or workbook.

### Secondary measures

Self efficacy at baseline showed a mean score of 5.88 out of 10, SD = 2.38. Those who participated in the intervention demonstrated a significant improvement in SE whencompared to the control group (Current effect: F(3, 216) = 4.37, *p* < 0.01). This difference was observed at week six by between group differences of M = 1.72, SD = 1.51, *p* = 0.03, ES = 0.76, 95 % CI (0.28, 1.22) (Table 4). No other significant differences were noted at other time intervals.

**Table 4:** Secondary outcome scores

	Experimental Group *n* = 35	Control Group *n* = 39	Between-group difference over time
	*Mean* ± SD (out of 10)	*Mean* ± SD (out of 10)	*Mean* (95 % CI), ES (95 % CI)
Baseline Self-efficacy HRQoL	5.83 ± 2.57 0.36 ± 0.34	5.92 ± 2.24 0.38 ± 0.32	
Week 6 Self-efficacy HRQoL	7.36 ± 2.33* 0.65 ± 0.31	5.73 ± 1.96 0.44 ± 0.30	1.72(0.42, 3.0), ES = 0.76(0.28, 1.22) 0.23(0.6, 0.4), ES = 0.69(0.21, 1.15)
Week 12 Self-efficacy HRQoL	6.95 ± 2.30 0.60 ± 0.32**	5.51 ± 2.00 0.36 ± 0.35	1.53 (0.38, 2.7), ES = 0.67(0.3, 1.13) 0.26(0.06, 0.46), ES = 0.71(0.24, 1.18)
Month 6 Self-efficacy HRQoL	7.19 ± 2.12 0.55 ± 0.34	6.00 ± 1.93 0.37 ± 0.34	1.28(0.32, 2.2), ES = 0.59(0.12, 1.05) 0.2(0.05, 0.35), ES = 0.53(0.06, 0.99)

*indicates a significant improvement in SE of the experimental group compared to control group

**indicates a significant improvement in HRQoL of the experimental compared to control group

Health related quality of life of the sample at baseline was low (M = 0.37, SD = 0.33). The intervention group showed a significant improvement in HRQoL when compared to the control group (current effect: F(3, 216) = 4.45, *p* < 0.01). This difference was seen at week 12 by between groupdifferences of M = 0.26, SD = 0.58 *p* = 0.03, ES = 0.71, 95 % CI (0.24, 1.18) (Table 4). No significant differences wererecorded for EQ5D health state at any time point (Current effect: F(3, 216) = 2.47, *p* > 0.05).

Disability according to the HAQ disability index (DI) at baseline illustrated a moderate level of disability (M = 1.12, SD = 0.69). No significant differences were observed between groups for the DI during the study (Current effect: F(3, 216) = 8.51, *p* < 0.01) or for the HAQ pain visual analogue scale (VAS) during the study (Current effect: F(3, 120) = 5.97, *p* < 0.01). Additionally, there were no significant differences between groups in any of the tests from the physical performance task battery (normal walk, fastest 15 m test, six minute walk test, forward reach, upward reach, sock test and sit to stand). Further information on secondary measures is available from the authors.

Of the entire sample, 10 % were absent at week sixfollow up; six from the control group and two from the intervention group. At week 12, a higher attrition rate was seen with 23 % of the sample being absent for follow up measures; 12 from the control and six from theexperimental group. At month six, a further 2 % of the sample was absent at follow up measures; nine from the experimental group and ten from the control group. See Table 5 for details.

**Table 5:** Attrition rate details

	Week 6		Week 12		Month 12	
Reason for absence	Experimental	Control	Experimental	Control	Experimental	Control
Withdrawing from study	1	1	2	2	2	2
Falling ill	1	2	2	1	3	1
Forgetting		3		4		3
Transport			1			2
Receiving surgery			1	2	3	2
Work responsibility				2		
Other: funeral					1	
Total	2	6	6	11	9	10
Percentage of entire sample	10 %		23 %		25 %	

The results of this study presented here support the use of a six-weekphysiotherapist-led exercise and education programme to improve pain, in patients with osteoarthritis of the hip and/or knee awaiting arthroplasty. This is seen by clinically relevant findings in the experimental group with significant improvements in pain severity and interference with moderate to large effect sizes, when compared to the control group.

## Discussion

As pain is the most common complaint of those suffering with OA [67, 68], most studies performed in this field assess pain severity. In this study, the BPI shows an exercise and education intervention can result in amoderate effect on pain; as seen by the sustained significant reduction in pain severity scores over time. It is noted that the changes in pain severity between groups approach what is considered a meaningful difference in pain (3 on a scale of 0 – 10). The intervention’s reported mean effect size on pain (ES 0.94 and 0.74 at week 6 and month 6 respectively) is larger than previous studies reporting the effects of exercise *alone* on pain in persons with OA (ES 0.39 – 0.58) [69–72]. Fransen and colleagues similarly reported a large ES for pain after an exercise intervention in persons with knee OA (ES = 0.94, *p* < 0.01) yet combined data for hip and knee OA show small to moderate effect with a smaller effect at its lower CI (ES 0.46, 95 % Cl (0.35, 0.57) [71]. This is better matched to the lower confidence interval noted in the current study for reduction in pain severity. However, comparison to Fransen et al.s’ study on exercise should be made with caution as they only followed their participants for four months [71].

It is important to consider the precision of the reported effect size in this current study when interpreting results as the CI of 0.45 – 1.41 and 0.26 – 1.2 at week six and month six show a considerable difference inconfidence limits. The lower limit (0.45 and 0.26) of the effect size for changes seen in pain severity at week six and month six report a small to moderate effect at most whereas the upper limit (1.41 and 1.2) are seen as very large effects. Thus these results for pain severity should be interpreted with a degree of caution.

Elsewhere, effect sizes for pain severity in prior studies on similar integrated interventions in knee OA range from 0.2 to 0.27 [30, 73]. A study of integrated exercise and education by Lamb et al. [29] is more in line with the longer waiting periods (over a year) experienced in the SA population and is supported by the current study’s findings. Lamb et al. showed moderate effect sizes for pain improvement are attainable in those with knee OA in patients with symptoms on average of 10 years [29]. However, their study did not make use of a control group which highlights a strength of the current study’s design.

For a chronic pain condition such as OA, it is not only how much pain one has but the debilitating effect that pain has on the person’s daily life and interactions that requires attention [74]. An intervention including self-management strategies, such as the present intervention, with a focus on the effect of pain rather than just the amount of pain is recommended [74]. Previous research has shown the debilitating effects of pain from being on relatively short waiting lists (less than 12 months); with worse pain and effects on function being correlated to longer periods of waiting [75, 76]. As a result of this sample’s long waiting period (average of two years, six months), it was anticipated that this group would present at baseline with severe interference in physical and emotional functioning as a result of pain (M = 6.23, SD = 2.25). Despite this, the findings show that the intervention can significantly reduce and sustain lower pain interference scores.

Again, the large mean effects in pain interference,particularly at week six (ES = 1.2, 95 % CI, 0.7, 1.69) and month six (ES = 0.98, 95 % CI, 0.49, 1.45), are notably better than the ES of pharmacological agents such as NSAIDS (ES = 0.32, 95 % CI 0.24, 0.39) and acetaminophen (ES =0.21, 95 % CI, 0.02, 0.41) for pain in people with OA [77]. Both NSAIDs and analgesics are prescribed to most, if not all, patients on a waiting list as part of the first-line management of OA despite small ES [9]. Furthermore, the ES of the combined treatment of exercise and education used in this study on pain interference was again larger than the ES on pain in hip and knee OA for aerobic exercise *alone* (ES = 0.52, 95 % CI, 0.34, 0.70), strength exercise *alone* (ES = 0.32, 95 % CI, 0.23, 0.42), water-based exercise *alone* (ES = 0.25, 95 % CI, 0.02, 0.47) or education or self-managementinterventions *alone* (ES = 0.06, 95 % CI, 0.02, 0.10) [77]. Therefore, it can be said that the effects of change for pain interference are of a higher precision than thosereported for pain severity with the differences in CI limits being smaller, particularly for pain interference atweek 6. The large and more precise effect of the combined intervention seen in this study for pain interference appear more accurate and may be considered a more effective treatment strategy for these patients than pharmacological agents or individual treatment approaches.

The individual answers grouped together formed themes which was helpful to link the benefits observed in pain scores over time with responses given relating to pain relief. Many (53 %) of the participants’ responses to the questionnaire supported the significant improvement in pain scores seen across the sample.

The participants’ improved self efficacy after six weeks shows that the intervention may have resulted in improved self-belief in their ability to achieve goals orperform an activity. However, the ES is moderate and the significant difference between groups is quite small (M = 1.72, SD = 1.51) with a wide CI limit (0.28, 1.22) and the effect is not sustained beyond six weeks. This finding should be interpreted with caution.

The significant improvement in HRQoL at week 12 may be interpreted as a consequence of improvements in pain levels and interference since commencement of the study. However this finding should also be interpreted with caution as correlation calculations were not performed to confirm this. Similarly as mentioned above, even though the ES is moderate, the CI limits are again very wide (0.24, 1.18) and this significant difference is not consistent at week six or sustained at month six.

The findings of no improvements in disability or function are not necessarily surprising in this sample awaiting surgery with moderate to high levels of disability at baseline. It is anticipated that a more meaningful improvement in pain severity and interference could possibly translate into improvements in these areas and further research could confirm this hypothesis.

Since the wait for surgery is so lengthy in the public health sector in SA, the benefits recorded after this short intervention are encouraging and support the previously identified need to improve pain and related function in those whose waiting period is longer than a year [22]. The current study appears to have begun to meet this need by reducing pain severity and interference while awaiting surgery after just six weeks of an exercise and education intervention. This is in contrast to otherstudies which report benefits to be evident only after a longer period of time [23, 78]. Despite the current study following participants for shorter than the recommended duration of 12 months, the results are considered as a positive contribution towards further research in this field as improvements in pain severity and particularly pain interference were observed after only six weeks and sustained at month six.

### Strengths/limitations

A moderate sample was recruited from two different regions of the country, servicing two populations with osteoarthritis. This allows for a limited degree of generalizability to the broader population of OA sufferersawaiting a joint replacement, in the SA public health system. However, it is important to note that over 90 % of those on the waiting lists were not included in the study for different reasons (Fig. 1). This is a potential source of recruitment bias. Those not included in the study for unspecified reasons may have been either (a) too ill to participate or (b) not experiencing sufficient pain to be motivated to participate. If the majority of unspecified non-participants fell into either of these categories, the results may be biased. Another aspect is that this sample had slightly low educational levels compared to those in upperincome countries; which is representative of the large portion of the previously disadvantaged population, who consequently also have low income levels and therefore attend public health facilities [79]. Caution should be applied as these findings might not be transferable to those in the private health sector who may have higher educational levels or wait shorterperiods for surgery.

Although the use of medication and percentage painrelief obtained from medication was recorded on the BPI, specific use of medication in terms of type, dose or how often medication was used was not recorded. This could be valuable data to determine whether the interventionaffected the use of medication or whether medication use influenced how participants responded to the intervention. It is recommended that future research into this field should consider medication use and effects thereof.

As Hurley and colleagues state, loss to follow up is a common problem during research [32]. This was not too concerning at the first follow up session as seen by only 10 % loss to follow up, however higher attrition rates at week 12 (23 %) and month 6 (25 %) should be considered during interpretation of the findings. To manage missing data, data was carried forward from the lastobserved measure. This may have led to blunting ofresults and therefore it is recommended that for future studies researchers explore a different approach such as “multilevel modeling and multiple imputation to generate robust predictions of the effect of missing data” [80]. Despite the risk of further loss to follow up, it is recommended that future trials extend the follow up period to at least 12 months as long lasting benefits are especially needed in persons on a long surgical waiting list [24].

Another study is currently underway to extend follow up duration which will provide valuable comparative data whereby the current findings can be compared with prior long term research concerning persons with OA awaiting arthroplasty as well as post-operatively. In addition to this, it is suggested that future researchevaluates whether such an intervention has differenteffects on those with OA of the knee or hip, and/or any effect on the need to still be on a waiting list for surgery.

## Conclusion

The rationale behind undertaking the present study was primarily due to the rising burden of osteoarthritis affecting thousands of individuals awaiting surgery in SouthAfrica. This study provides evidence for an acceptable and effective evidence-based short term non-pharmacological and non-surgical intervention that can be easily delivered by a physiotherapist. The intervention appears to beeffective in reducing this population’s suffering by reducing pain severity and interference and is well received by patients. Further research into this population of interest will be of value in determining longer lasting effects while awaiting surgery, whether the need for surgery can berevised, whether medication use has any influence as well as whether such a pre-operative intervention can have any significant effects post-operatively.

## Abbreviations

ACR, American College of Rheumatology; ACSM, American College of Sports Medicine; ANOVA, analysis of variance; BMI, body mass index; BPI, brief pain inventory; CI, confidence interval; CONSORT, Consolidation of Standards of Reporting Trials; DI, disability index; ES, effect size; ESCAPE, enabling self-management and coping with arthritic knee pain through exercise; HAQ, health assessment questionnaire; ICF, International Classification of Functioning, Disability and Health; IMMPACT, initiative on methods, measurement, and pain assessment in clinical trials; OA, osteoarthritis; SA, South Africa; VAS, visual analogue scale

## Additional file

Additional file 1: “Appendix” includes examples of the exercise component of the intervention, the Brief Pain Inventory, open-ended questionnaire, secondary measures and the CONSORT guidelines table. (DOCX 115 kb)
