# Supplementary material for: Significant improvements in pain after a six-week physiotherapist-led exercise and education intervention, in patients with osteoarthritis awaiting arthroplasty, in South Africa: a randomised controlled trial
Source: BMC Musculoskelet Disord. 2016 May 27;17:236. doi: 10.1186/s12891-016-1088-6 (PMC4884378; doi:10.1186/s12891-016-1088-6)
Supplement: Additional file 1: — “Appendix” includes examples of the exercise component of the intervention, the Brief Pain Inventory, open-ended questionnaire, secondary measures and the CONSORT guidelines table. (DOCX 115 kb) [file 12891_2016_1088_MOESM1_ESM.docx]

**Appendix**

***An exercise routine***

This is a 20 – 30 minute exercise routine which is safe for people living with osteoarthritis. This routine includes exercises which make you stronger (strength exercises), more flexible (stretching exercises) and fitter (endurance exercises).

1. Start by standing up straight and tall, feel your weight across your feet, relax your shoulders and open your chest, hold your head straight. Take a deep breath in and breathe out.

2. March on the spot for 2 minutes. March at a steady pace – that is a pace which you can maintain for 2 minutes. Do not start fast and get slower or start slowly and get faster. Pace yourself, start and finish at the same speed. You should be marching so that you can feel you are breathing a little bit harder than normal, you should be able to talk but not be able to sing.

3. Now stretch your neck – keep your shoulders relaxed and turn to look over your right shoulder – hold it for 20 seconds. Bring your head back to the middle, then turn to look over your left shoulder – hold it for 20 seconds and then bring your head back to the middle. Now put your left ear on your left shoulder - hold it for 20 seconds and then bring your head back to the middle. Repeat to the right. Now put your chin on your chest - hold it for 20 seconds and then bring your head back to the middle.

4. Roll shoulders forwards 5 times, then roll your shoulders backwards 5 times. Then stretch your arms by stretching your right arm across your body to the left and holding for 20 seconds and then repeat with the other arm.

5. March on the spot for another 2 minutes – 30 steps normal, 30 steps lift your knees up as high as you can. Keep changing every 30 steps.

6. Stretch your quadriceps muscles by bending your right leg backwards and holding your foot if possible below your buttock. You will feel the stretch down the front of your thigh. Hold it for 20 seconds and then do the same on the left.

7. Stretch your hamstring muscle by putting your right heel on the ground and pulling your toes upwards, put your hands above your knee and lean forward to feel the stretch behind your knee. Repeat on the left for 20 seconds too.

8. Sit on a chair – make both your knees straight and then bend again. Do this 30 times. This works your front thigh muscles.

9. Then with your arms folded on your chest, stand up from the chair and sit down again. Keep sitting down and standing up for 2 minutes. Do this at a steady pace – that is a pace which you can maintain for 2 minutes.

10. March on the spot for 2 minutes – 30 steps normal, 30 steps lift your feet up as high as you can (try to kick your buttocks). Keep changing every 30 steps.

11. Stand on one leg at a time for 30 seconds each: use support by putting your hand on a wall or chair if necessary but try balance without holding on. Do this twice on each leg

12. Step ups: Step onto and off of a low step for 2 minutes. Start with the right leg and then step up with the left leg and alternate. This works both your front and back legs muscles as well as your hip muscles.

13. March on the spot for 2 minutes – 30 steps normal, 30 steps lift your knees up as high as you can. Keep changing every 30 steps.

14. End off the session by stretching your trunk: Slide your hands down the front of your thighs to see if you can touch your toes. Bend sideways by sliding your hand down the side of your leg. Hold this for 30 seconds and then do it to the other side.

Finish by standing up straight and tall, feel your weight across your feet, relax your shoulders and open your chest, hold your head straight. Take a deep breath in and breathe out.

Example of exercise regimen and possible progression (Hurley, M. et al, 2007)

| **Exercise** | **Aim** | **Activities** | **Progression** |
| --- | --- | --- | --- |
| Walking | General warm up  Improve cardiovascular fitness | Walking on the spot for 2 minutes | Increase speed and duration  Incorporate arm swings and boxing  Lift knees higher |
| Quadriceps Strength | Improve strength | Sitting with knees at 90, extend the knee. | Increase speed and repetitions  With back against a wall, flex hips and knees into a squat |
| Standing on one leg | Balance and coordination | Single leg stance, supported by hand on a wall if necessary | Increase duration  Stand on soft matt  Remove wall support |
| Sit to stand | Strength, control, function | With arms folded, sit to stand from chair or bench | Decrease height of chair  Increase reps |
| Step ups | Strength, control, function | With one foot on step, step on/off step | Increase height of step  Increase repetitions |
| Stretches | Range of motion and flexibility | Stretches of major upper and lower limb muscles groups: neck, shoulder, arms, torso, legs and feet | N/A |
| Interactive Activities | Social and fun, cardiovascular, balance, concentration , coordination | Large gym-ball bouncing between participants. Stepping/dancing activities | Use smaller ball and throw to each other.  Stand in line and pass ball backwards |

**Brief Pain Inventory**

1.
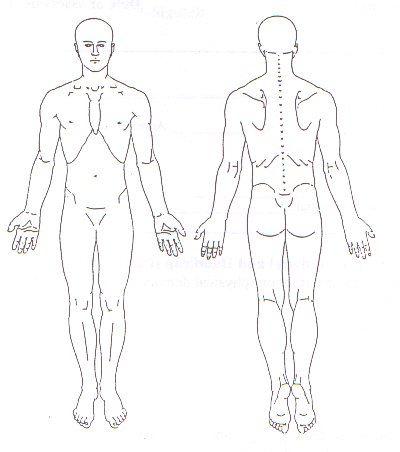
Throughout our lives, most of us have had pain from time to time (such as minor headaches, sprains, and toothaches). Have you had pain other than these everyday kinds of pain during the last week?

Yes No

1. On the diagram, shade in the areas where you feel pain. Put an **X** on the area that hurts the most.
2. Please rate your pain by circling the one number that best describes your pain at its ***worst*** in the last week.

0 1 2 3 4 5 6 7 8 9 10

No Pain as bad as

Pain you can imagine

1. Please rate your pain by circling the one number that best describes your pain at its ***least*** in the last week.

0 1 2 3 4 5 6 7 8 9 10

No Pain as bad as

Pain you can imagine

1. Please rate your pain by circling the one number that best describes your pain on the ***average.***

0 1 2 3 4 5 6 7 8 9 10

No Pain as bad as

Pain you can imagine

1. Please rate your pain by circling the one number that tells how much pain you have ***right now.***

0 1 2 3 4 5 6 7 8 9 10

No Pain as bad as

Pain you can imagine

1. What treatments or medications are you receiving for your pain?

____________________________________________________________________________________________________________________________________________________________________________________

1. In the last week, how much ***relief*** have pain treatments or medications provided? Please circle the one percentage that most shows how much *relief* you have received.

0% 10% 20% 30% 40% 50% 60% 70% 80% 90% 100%

No Relief Complete relief

1. Circle the one number that describes how much, during the past week, **pain** has ***interfered with*** your:
2. ***General Activity***

0 1 2 3 4 5 6 7 8 9 10

Does not interfere Completely interferes

1. ***Mood***

0 1 2 3 4 5 6 7 8 9 10

Does not interfere Completely interferes

1. ***Walking Ability***

0 1 2 3 4 5 6 7 8 9 10

Does not interfere Completely interferes

1. ***Normal Work*** (includes both work outside the home and housework)

0 1 2 3 4 5 6 7 8 9 10

Does not interfere Completely interferes

1. ***Relations with other people***

0 1 2 3 4 5 6 7 8 9 10

Does not interfere Completely interferes

1. ***Sleep***

0 1 2 3 4 5 6 7 8 9 10

Does not interfere Completely interferes

1. ***Enjoyment of life***

0 1 2 3 4 5 6 7 8 9 10

Does not interfere Completely interferes

CONSORT guidelines and indication of where items were reported in the manuscript

| Section/Topic | Item No | Checklist item | Reported in |
| --- | --- | --- | --- |
| Title and abstract | 1b | Identification as a randomised trial in the title  Structured summary of trial design, methods, results, and conclusions | Title  Abstract |
| Introduction  Background/objective | 2a  2b | Scientific background and explanation of rationale  Specific objectives or hypotheses | Abstract and background pg. 4 - 5  Background pg. 5 |
| Methods | | | |
| Trial design | 3a  3b | Description of trial design (such as parallel, factorial) including allocation ratio  Important changes to methods after trial commencement (such as eligibility criteria), with reasons | Methods: study design pg. 6 and recruitment procedure pg. 7 - 8  NA |
| Participants | 4a  4b | Eligibility criteria for participants  Settings and locations where the data were collected | Methods: participants pg. 6  Methods: intervention pg. 8  Figure 1 |
| Interventions | 5 | The interventions for each group with sufficient details to allow replication, including how and when they were actually administered | Methods: Intervention pg. 8 - 10  Appendix: example of exercise programme  Table 1  Reference 41 for educational workbook |
| Outcomes | 6a  6b | Completely defined pre-specified primary and secondary outcome measures, including how and when they were assessed  Any changes to trial outcomes after the trial commenced, with reasons | Methods: instrumentation pg. 10 – 11  Appendix: BPI |
| Sample size | 7a  7b | How sample size was determined  When applicable, explanation of any interim analyses and stopping guidelines | Methods: participants pg. 7 |
|  |  |  |  |
| Randomisation:  Sequence generation | 8a  8b | Method used to generate the random allocation sequence  Type of randomisation; details of any restriction (such as blocking and block size) | Methods: recruitment procedure pg. 7 – 8 |
| Allocation concealment mechanism | 9 | Mechanism used to implement the random allocation sequence (such as sequentially numbered containers), describing any steps taken to conceal the sequence until interventions were assigned | Methods: recruitment procedure pg. 7 |
| Implementation | 10 | Who generated the random allocation sequence, who enrolled participants, and who assigned participants to interventions | Methods: recruitment procedure pg. 7 - 8 |
| Blinding | 11a  11b | If done, who was blinded after assignment to interventions (for example, participants, care providers, those assessing outcomes) and how  If relevant, description of the similarity of interventions | Methods: recruitment procedure pg. 7 |
| Statistical methods | 12a  12b | Statistical methods used to compare groups for primary and secondary outcomes  Methods for additional analyses, such as subgroup analyses and adjusted analyses | Methods: statistical analysis pg. 11 – 12 |
| Results | | | |
| Participant flow (a diagram is strongly recommended) | 13a  13b | For each group, the numbers of participants who were randomly assigned, received intended treatment, and were analysed for the primary outcome  For each group, losses and exclusions after randomisation, together with reasons | Methods: recruitment procedure pg. 8  Figure 1  Discussion: strengths and limitations pg. 17 |
| Recruitment | 14a  14b | Dates defining the periods of recruitment and follow-up  Why the trial ended or was stopped | Methods: recruitment procedure pg. 7 – 8  Figure 2 |
| Baseline data | 15 | A table showing baseline demographic and clinical characteristics for each group | Included in main dissertation, available on request |
| Numbers analysed | 16 | For each group, number of participants (denominator) included in each analysis and whether the analysis was by original assigned groups | Results pg. 12  Included in main dissertation, available on request |
| Outcomes and estimation | 17a  17b | For each primary and secondary outcome, results for each group, and the estimated effect size and its precision (such as 95% confidence interval)  For binary outcomes, presentation of both absolute and relative effect sizes is recommended | Results pg. 13 – 14  Figures 3 and 4  Table 2 and 3 |
| Ancillary analyses | 18 | Results of any other analyses performed, including subgroup analyses and adjusted analyses, distinguishing pre-specified from exploratory | Full results of other outcomes not presented in this paper are available in the main dissertation on request |
| Harms | 19 | All important harms or unintended effects in each group | None reported |
| Discussion | | | |
| Limitations | 20 | Trial limitations, addressing sources of potential bias, imprecision, and, if relevant, multiplicity of analyses | Discussion: strengths and limitations pg. 17 |
| Generalizability | 21 | Generalizability (external validity, applicability) of the trial findings | Discussion: strengths and limitations pg. 17 |
| Interpretation | 22 | Interpretation consistent with results, balancing benefits and harms, and considering other relevant evidence | Discussion pg. 14  Conclusion pg. 18 |
| Other information | |  | |
| Registration | 23 | Registration number and name of trial registry | Pan African Clinical Trial Registry PACTR201409000885765 and PACTR201507001186115 |
| Protocol | 24 | Where the full trial protocol can be accessed, if available | N/A |
| Funding | 25 | Sources of funding and other support (such as supply of drugs), role of funders | Acknowledgements pg. 20 |
